# Tailored Pre-Operative Antibiotic Prophylaxis to Prevent Post-Operative Surgical Site Infections in General Surgery

**DOI:** 10.3390/antibiotics13010099

**Published:** 2024-01-19

**Authors:** Mason Vierra, Mohsen Rouhani Ravari, Fatemeh Soleymani Sardoo, Benjamin D. Shogan

**Affiliations:** 1 Pritzker School of Medicine, The University of Chicago, Chicago, IL 60637, USA; mason.vierra@uchicagomedicine.org; 2 Department of Surgery, The University of Chicago Medicine, Chicago, IL 60637, USA; mohsen@uchicago.edu (M.R.R.); t-9fsole@uchicago.edu (F.S.S.)

**Keywords:** surgical site infection, antibiotics, prophylaxis, surgery

## Abstract

The average American today undergoes three inpatient and two outpatient surgical procedures during one’s life, each of which carries with it a risk of post-operative infection. It has long been known that post-operative infections cause significant morbidity in the immediate peri-operative period, but recent evidence suggests that they can have long-term consequences as well, increasing a patient’s risk of infectious complications in unrelated surgeries performed months or even years later. While there are several theories on the origin of this association, including bacterial colonization of a post-operative infectious wound site, antimicrobial resistance from curative courses of antibiotics, subclinical immunosuppression, or the creation of an inflammatory “pathobiome” following an infectious insult, it is ultimately still unclear why patients who experience a single post-operative infection seem to be at a significantly higher risk of experiencing subsequent ones. Regardless, this association has significant implications for the routine use of pre-operative antibiotic prophylaxis. Indeed, while the prescription of antibiotics pre-operatively has dramatically reduced the rate of post-operative infections, the chosen prophylaxis regimens are typically standardized according to national guidelines, are facing increasing antimicrobial resistance patterns, and have been unable to reduce the risk of post-operative infection to acceptably low levels for certain surgeries. As a result, some clinicians have speculated that tailoring pre-operative antibiotic prophylaxis according to a patient’s prior infectious and operative history could improve efficacy and further reduce the rate of post-operative infections. The purpose of this review is to describe the evidence for the link between multiple post-operative infections and explore the efficacy of individualized pre-operative prophylaxis.

## 1. Introduction

Americans today undergo more surgeries, for a wider variety of indications, than ever before. Each operation that a patient undergoes carries with it a risk of post-operative infection, a complication that may be mitigated, though not eliminated, through pre-operative antibiotic prophylaxis. While it has traditionally been assumed that a successfully treated post-operative infection has little bearing on the risk profile of a patient undergoing surgery in the future, newer studies have linked prior post-operative infections with subsequent ones, even if the surgeries are unrelated or performed many months or even years apart. This would suggest a role for individualized pre-operative prophylaxis based on a patient’s prior infectious history. The purpose of this review is to describe the literature that both supports and refutes this approach and provide context on the origin of the association behind multiple post-operative infections.

To write this review, we performed a literature search for studies published between 1960 and 2023 using the Google Scholar and PubMed databases. This search was conducted between 1 December 2023 and 31 December 2023 by MV, MR, and SR. The keywords used were surgical site infection (SSI), antibiotics, prophylaxis, reoperation, surgery, and post-operative infections. We excluded non-English journal articles, case studies, and articles in which the full text was not available. We included English-language articles published between 1 January 1960 and 1 December 2023 in which the full text was available and the study population was greater than 10 patients. Our initial search produced 252 studies, of which 123 were included in the final article. 

## 2. The Rates and Risks of Reoperation in General Surgery

In 2021, the average life expectancy in the United States was 76.1 years, a dramatic increase from 47 years at the turn of the 20th century [1]. This increase in life expectancy, along with the growth of the American healthcare industry from a USD 27.1 billion industry (5% of GDP) in 1960 to a USD 4.25 trillion industry (18.3% of GDP) in 2021, has resulted in Americans interacting with medical professionals at unprecedented rates [2,3]. Similar trends have occurred worldwide, with global life expectancy having risen from 32 years in 1900 to 71 years in 2021, and with medical care having become more accessible to a wider swath of the global population than ever before [4,5]. 

This is particularly true of surgical care. Once considered an effort of last resort, surgery became safer in the mid-1900s with the advent of anesthesia, sterile operating techniques, and antibiotics. In the intervening decades, improvements in surgical techniques and technology have allowed more and more people worldwide to be offered surgery for a wider variety of procedures [6]. Surgery has become especially accessible in developed countries such as the United States, and Lee and Gawande estimate that today, the average American undergoes approximately three inpatient and two outpatient surgical procedures over one’s life [7,8]. This will likely increase in the future, as life expectancy is projected to reach over 80 years by 2050, surgical technology is continually improving, and patients are increasingly willing and able to undergo procedures in the later years of their lives [9,10,11,12]. 

Importantly, Lee and Gawande’s findings imply that some Americans undergo multiple surgeries during their lives. While these surgeries may be entirely unrelated from one another, a certain portion may be considered “repeat operations” or “reoperations”, performed for same prior pathology or in response to a complication from a prior related operation. While this risk varies based on several factors, patients undergoing abdominal surgeries are particularly likely to require subsequent operations. In the 30-day post-operative period, abdominal surgeries can be complicated by bleeding, infection, dehiscence, anastomotic leak, bowel injury, and obstruction, often requiring reinterventions such as laparotomy, washout, ostomy creation, bowel resection, or fascial closure. This risk appears to be lowest in the pediatric population, with anywhere from 0.4% to 4.3% of general pediatric surgery operations requiring an unplanned reoperation, and highest in the emergent or traumatic setting, with greater than one-third of patients undergoing emergent laparotomy ultimately requiring relaparotomy [13,14,15]. Broadly speaking, the 30-day reoperation rate amongst all elective general surgery procedures is approximately 7.5% [16,17,18,19]. 

After this immediate post-operative period, patients may require repeat surgery for a variety of reasons, such as malignant disease recurrence, hernia repair, ostomy relocation or closure, or emergent operative repair of a bowel obstruction often caused by adhesions from the index abdominal surgery. Due to these risks, 12% to 20% of patients are expected to require an additional abdominal surgery within two years of an index abdominal surgery, and within four years, the risk of reoperation jumps to 27%, with nearly 10% undergoing multiple operations [20,21]. As a case in point, 36–66% of elective abdominal surgeries performed today are reoperations [22,23,24]. 

## 3. The Link between Recurrent Post-Operative Infections

Each of these operations carries certain risks, one of which is post-operative infection or surgical site infection (SSI). In the United States, SSIs make up approximately 20% of all hospital-acquired infections and complicate 2–5% of inpatient surgical procedures, with a total annual incidence of approximately 500,000 [25,26]. Surgeries of the abdomen are especially high-risk to result in a post-operative infection, primarily due to the high bacterial load in the large bowel [26,27]. This is significant because SSIs are the most common cause of readmission after surgery, extend hospital length of stay, cost the U.S. healthcare system up to USD 10 billion annually, and increase the risk of ICU admission and mortality [28,29]. 

While the microbiological characteristics of SSIs vary based on procedure type and hospital setting, the responsible organism is usually from the patient’s own flora [30]. Staph aureus and coagulase-negative Staphylococci are the most isolated organisms, but anaerobes and Gram-negative bacilli such as Enterococcus species, Enterobacteriaceae species, Pseudomonas aeruginosa, and Escherichia coli are often implicated in SSIs following surgeries of the gastrointestinal tract [31,32]. In recent years, organisms isolated from SSIs have shown increasing resistance to commonly used antibiotics such as methicillin, carbapenem, vancomycin, and beta-lactams [33]. Because SSIs are typically treated with antibiotic therapy (with or without debridement, drainage, or wound exploration), these resistance patterns have raised concerns that routine antibiotic regimens will be increasingly rendered inadequate [34].

Various models and indices have been implemented to pre-operatively predict a patient’s risk of SSI, including the National Nosocomial Infections Surveillance (NNIS) risk index, the COLA (contamination, obesity, laparotomy, American Society of Anesthesiologists (ASA) grade) score, and the National Healthcare Safety Network (NHSN) multivariable risk model [35,36]. While these models include many well-validated metrics and have shown some predictive value, a significant portion of SSIs still arise unexpectedly.

One metric that has not been included in these risk models is a prior history of SSI, as it has traditionally been assumed that a prior post-operative infection, if treated effectively, has little bearing on a patient’s risk of infectious complications following subsequent procedures, especially if the operations are unrelated or separated by an extended period. Yet, in the past several years, growing evidence has emerged that even a single post-operative infection increases the risk of infectious complications well after the index procedure. 

Some of the first studies to suggest this were published in the 1960s–1980s. Investigating recurrent and delayed wound infections following hernia repairs and other abdominal surgeries, several studies found that a wound that was the site of a previous post-operative infection, even if effectively treated, could be colonized by viable bacteria capable of reemerging to cause infections in subsequent operations [37,38]. Furthermore, it was found that incisional hernia repair of a previously infected wound placed patients at a significantly higher risk of reinfection compared to patients undergoing similar procedures without an infectious history, even if the initial infection had been completely eradicated and the wound had completely healed [39]. Subsequent studies found that not only was SSI the most common cause of reoperation following ventral hernia repair, but that a prior soft tissue infection, even one that arose more than one year in the past, significantly increased a patient’s risk of SSI following ventral hernia repair [40,41]. On a bacterial level, a recent study found that a significant portion of reinfections of hernia mesh are caused by the same microbes, confirming the findings of bacterial colonization and reemergence described in earlier studies [42]. These findings have prompted the Ventral Hernia Working Group to suggest that a prior wound infection should be considered a significant risk factor for SSI in patients undergoing ventral hernia repair [43]. 

Faraday et al.’s 2013 study broadened the scope of investigation. In their prospective cohort study of 613 patients undergoing cardiac, vascular, cranial, and spinal surgery, patients with a prior history of skin infection requiring antibiotic therapy had a significantly increased risk (adjusted estimates ranging from 2.5-fold to 3.5-fold) of developing a post-operative SSI or experiencing infectious death, even when controlling for traditional SSI risk factors and even in patients with adequate adherence to antiseptic practices [44]. Importantly, whether the soft tissue infection occurred within the year of surgery or in the distant past did not meaningfully affect this association. The authors speculated that a prior skin infection could be evidence of a susceptible state that made seemingly immunocompetent patients more likely to experience a post-operative infection. 

Several years later, Cohen et al. published similar findings. Concerned with the risk of antimicrobial resistance caused by pre-operative antibiotic prophylaxis, these investigators retrospectively reviewed the cases of 689 patients who developed an SSI following elective surgery [45]. Nearly half (49%) of patients had SSIs that were antibiotic-resistant. Further, the authors found that the primary predictor of a post-operative resistant infection was a prior culture-proven infection, and they argued that prior individual infections should be considered when selecting an antibiotic prophylaxis pre-operatively. 

O’Brien et al. noted that a post-operative infection was associated with an increased risk of a subsequent infection and worse overall outcomes [46]. In their retrospective cohort study of 659,486 patients, those who developed a 30-day post-operative infection, the most common of which was an SSI, had a higher incidence of infection and mortality on post-operative days 31–365 compared to patients who did not have an initial post-operative infection. In fact, patients with a 30-day post-operative infection had a 3.2-fold higher risk of 1-year infection compared to those who did not. 

Guidry et al. tied recent antimicrobial exposure to SSI risk by assessing the recent antimicrobial exposure of patients undergoing elective surgeries [47]. Amongst 1538 patients, 34.1% had received antimicrobial treatments in the prior three months, an incidence consistent with previously reported data [48]. Compared to the unexposed cohort, these patients had higher rates of any complication, infection, and SSI, a longer median hospital stay, and a higher rate of return to the operating room. Colorectal surgery patients had the highest exposure rate (71.7%), but whether these patients were excluded or included from the analysis, antimicrobial exposure was still independently associated with a higher risk of any infection or any complication. The authors assumed that the exposed cohort had received antibiotics for a prior infection and speculated that one of these exposures—either the infection or the curative antibiotics—had resulted in an inflammatory or immunosuppressed state that made them susceptible to post-operative infections.

Riccio et al. demonstrated similar findings in their investigation of whether a longer duration of antibiotics for the treatment of an intra-abdominal infection (IAI) increased the risk of a subsequent extra-abdominal infection (EAI) within the same hospitalization [49]. Reviewing 2552 cases of IAIs, the 21.5% of patients who went on to develop an EAI had a significantly increased initial duration of antibiotic therapy for treatment of their IAI compared to patients who did not develop EAI. Further, the rate of EAI following IAI was just 13.3% in patients treated with a <7 day course of antibiotics compared to 25.1% in those who underwent at >7 day course. The investigators noted that while this relationship could have simply been due to the selection of resistant bacteria, which is a well-known association, it also could have been attributable to less well-understood changes in the gut flora or immune system. While this study described two infections within the same hospitalization, as opposed to the other studies discussed in this review that have linked infectious episodes separated in time, it nonetheless provides evidence that the initial insult of an SSI, and the chosen antibiotic treatment, can have some bearing on subsequent infectious risk.

Furthermore, a recent cross-sectional study by Khan et al. investigating the prevalence and etiology of SSIs following abdominal surgery found that a previous surgery was a significant risk factor for the development of an SSI [50]. Unfortunately, the authors did not describe the patients’ prior surgical history in detail, so it is unclear what these operations were and whether the patients suffered from infectious complications. Thus, while it is entirely possible that this association was due to a confounding variable that increased a patient’s likelihood of both requiring surgery and experiencing a post-operative SSI, it is also possible that something occurred at the time of their prior surgery to increase their long-term risk of post-operative infectious complications.

While these studies implicated prior surgeries, infections that selected for resistant organisms, and antimicrobial use, in subsequent post-operative infections, it was Feldt et al.’s 2022 study that tied these associations together [51]. Feldt et al. retrospectively studied the post-operative infection rate of 758 patients who had undergone two unrelated abdominal operations between 2012 and 2018. One hundred and fourteen (15%) patients developed an SSI after their first operation. Of these, 22.8% developed an infection after their second procedure compared to just 9.5% of patients who did not experience an SSI from their first procedure. In multivariable analysis, patients with a prior infection had 2.49 times greater odds of acquiring a second infection compared to patients who did not have a prior infection, with a mean duration of 366.4 days between procedures. The study’s microbial analysis implicated inadequate pre-operative prophylaxis and antimicrobial-resistant bacteria in this association. Infections after the second operation were more likely to be resistant to routine antibiotics compared to infections after the first procedure (82.3% versus 64.1%). Furthermore, nearly half (49%) of infections after the second procedure were resistant to the antibiotic prophylaxis chosen for surgery, and 84% of the patients affected had also experienced an antibiotic-resistant infection after their first operation. 

These most recent results are striking for several reasons. First, this study was one of the first with a large sample size to show that a previous SSI is an independent risk factor for a subsequent SSI. Second, this association was found regardless of whether the surgeries were related, and with a mean duration between procedures of greater than one year, perhaps suggesting a global susceptibility or systemic risk as opposed to a recurrent or inadequately treated local infection. Third, the microbial analysis suggested that the bacteria responsible for an initial SSI could be responsible for future SSIs, although this was not proven as a causative association. Finally, this study speculated that standard pre-operative prophylaxis may not only inadequately cover those who have suffered previous post-operative infections but may even induce colonization with antibiotic-resistant bacteria that can increase future post-operative infectious risk.

These findings described by Feldt et al. have significant implications for the population at large. One of the cornerstones of SSI prevention, and indeed safe surgery more broadly, is pre-operative antibiotic prophylaxis. Shown to reduce the risk of SSIs by as much as 40%, pre-operative prophylaxis regimens are now based on widely disseminated, evidence-based guidelines and have been adopted by nearly all surgeons in the United States [52,53]. However, recommended regimens are typically empirical, lacking detail on local antibiogram data or individual characteristics, and they certainly do not consider prior infectious history. Most importantly, they have significant room for improvement, as they have not been able to reduce the SSI rate to acceptably low levels in many general surgery operations [54,55]. Thus, with Americans living longer, undergoing more procedures, and experiencing significant antibiotic exposure during their lives, the possibility that standard pre-operative antibiotic prophylaxis may be less effective or induce antimicrobial resistance in the subset of the population with a prior surgical or infectious history raises questions about whether “one size fits all” guidelines need to be reconsidered. In short, in today’s world of multiple surgeries (each with its own SSI risk) and frequent exposure to antibiotics, Feldt et al.’s study suggests that it may be reasonable to individualize pre-operative prophylactic antibiotic regimens based on prior surgeries, prior infections, or microbial colonization and resistance.

## 4. Tailored Pre-Operative Antibiotic Prophylaxis

As far as we can tell, no studies have specifically assessed individualized prophylaxis based on prior SSI culture data. However, there is a strong precedent, in both the surgical and medical community, of tailoring antibiotics based on screening surveillance, risk categorization, or culture data from an event other than a prior SSI.

### 4.1. Precedent in Non-Post-Operative Infections

In the medical community, this approach has shown promise in the treatment of UTIs. MacFadden et al. showed the predictive utility of prior urine cultures in identifying the organism and susceptibility in subsequent urine cultures in their retrospective cohort study of 4351 patients with multiple positive urine cultures at different points in time [56]. Organismal correspondence and susceptibility correspondence were 57% and 83%, respectively, at 4–8 weeks, which were greater than what would be expected from chance alone, and in a follow-up study, the incorporation of individual patient characteristics and prior culture results into antibiogram-guided therapy improved predictions of antimicrobial susceptibility to empirical therapy [57]. Linsenmeyer et al. used prior culture data to improve the accuracy of empirical therapy for multidrug-resistant (MDR) UTIs [58]. In 95 patient episodes of MDR UTIs, 82% showed the same pathogen as the patient’s prior uropathogen. The chosen antibiotic provided effective coverage in 77% of cases when concordant with prior microbiological data versus just 33% when discordant with prior microbiological data, regardless of whether the prior UTI and the index UTI were caused by the same pathogen. In fact, choosing an antibiotic concordant with the prior microbiological data was associated with a seven-fold greater chance of effectively treating the index UTI. Importantly, the interval between episodes did not affect the accuracy of therapy. Khasawneh showed similar results when considering multiple infection types [59]. These investigators studied 970 episodes of recurrent bacterial infections (mostly UTIs, sepsis, pneumonia, and wound infections) and found that 65.1% of cultures between episodes matched and that the accuracy of empirical therapy was improved seven-fold when concordant with prior microbiological data. Finally, Frakking et al. showed that the accuracy of empirical antibiotic therapy was increased in bacteremic patients if they were known to be colonized with multidrug-resistant (MDR) bacteria and their empirical therapy was modified accordingly [60].

While these studies were conducted in the medical community, the data nonetheless support the use of tailored prophylaxis based on an individual’s prior culture and antibiotic sensitivity rather than the current prophylaxis guidelines that are agnostic to the individual’s prior history. 

### 4.2. Pre-Operative Screening and Tailored Prophylaxis

Within the surgical population, the most common example of targeted prophylaxis is in those who have been found to be colonized or infected with resistant bacteria on pre-operative screening. The best example of this involves MRSA. Increasing in incidence worldwide and one of the most common organisms isolated in SSIs, there is strong evidence that a documented prior MRSA colonization or infection is associated with a subsequent MRSA infection and an increased risk of SSI [44,61,62]. This relationship seems to be site-independent, with an MRSA infection of the urine, blood, or soft tissue conferring an increased risk of long-term SSI [41,61]. Fortunately, the empirical prescription of vancomycin, clindamycin, or other tailored regimens pre-operatively in patients colonized or infected with MRSA has shown efficacy in decreasing the overall SSI rate and the SSI rate due to MRSA [63,64]. Furthermore, the empirical prescription of broader-spectrum antibiotics in “high prevalence” institutions, in “high risk” patients, and in certain surgeries such as those involving prosthetic joint insertion or sternotomy, regardless of actual evidence of MRSA infection or colonization, has been shown to reduce post-operative infection rates [65,66]. Today, the use of tailored antibiotics based on MRSA colonization or in “high risk” circumstances is now recommended by many societal guidelines [67,68].

Resistant Gram-negative bacteria (GNB), such as extended-spectrum B-lactamase-producing enterobacter (ESBL-PE), have also garnered attention as potential targets for surveillance [69]. In studies of colorectal, hepato-pancreato-biliary (HPB), abdominal, and gynecological surgery, carriage prevalence of resistant GNB has been found to be as high as 18%, with prior antibiotic use, immunosuppressive therapy, biliary manipulation, and even abdominal surgery itself as notable risk factors [70,71,72]. These studies have associated colonization with a significantly increased risk of SSI, but importantly, several have demonstrated mitigating effects through pre-operative screening and modification of prophylactic regimens accordingly [70,71,72,73]. One of the first procedures in which this was tested was the transrectal ultrasound-guided prostate biopsy, which in the early 2000s had begun to show significant infectious complications from GNB resistant to standard fluoroquinolone prophylaxis [74,75]. Clinicians began to take rectal cultures pre-operatively to identify fluoroquinolone-resistant flora and modified their prophylaxis accordingly. This practice resulted in significant reductions in the risk of SSI in carriers of resistant GNB and is now considered routine in many settings [74]. In the orthopedic community, Núñez-Pereira et al. screened high-risk patients undergoing spinal surgery for GNB with pre-operative urine culture, and through tailored prophylaxis in patients with positive cultures, they were able to significantly reduce the SSI rate due to GNB and bring the overall SSI rate in line with the rates seen in “low-risk” patients who had received standard prophylaxis [76]. Similarly, Nutman et al. surveilled patients undergoing elective colorectal surgery for ESBL-PE colonization with a rectal swab [77]. Prescribing ertapenem as opposed to cephalosporin and metronidazole in patients with a positive swab, they significantly reduced the SSI rate and found a number needed to treat (NNT) of 13 and a number needed to screen (NNS) of 45–138. De Pestana et al. even demonstrated that a rectal swab for GNB highly correlated with bile culture in those undergoing pancreaticoduodenectomy, and speculated that a positive rectal swab could help direct prophylaxis [73]. 

In the transplant population, surveillance of resistant organisms with concomitant tailored prophylaxis has shown some promise as well. This is particularly important due to the high morbidity and mortality associated with post-operative infections with resistant organisms in such vulnerable, immunosuppressed patients. For example, Banach et al. used perirectal cultures to screen 61 patients undergoing liver transplantation for vancomycin-resistant enterococcus (VRE), a pathogen with particularly devastating effects in transplant patients [78]. VRE carriers were prescribed daptomycin in addition to standard prophylaxis. Post-operatively, just 3 of 27 VRE carriers developed VRE infections, a significantly lower rate than what has been described in other studies, and the authors concluded that pre-transplant VRE surveillance with targeted prophylaxis had likely provided a benefit [79]. Logre et al. and Bert et al. screened transplant patients with rectal swabbing and found high colonization rates with ESBL-PE [80,81]. While this was found to be a significant risk factor for post-operative infection with the same organism, thereby suggesting a benefit to targeted prophylaxis, the authors noted that fecal carriage alone was an imperfect prognostic indicator. As a result, they argued that fecal carriage should be used in conjunction with other known risk factors such as a previous infection with ESBL-PE, recent exposure to antibiotics, a history of spontaneous bacterial peritonitis, or MELD score > 25, to guide tailored prophylaxis regimens. Transplant societies today do not recommend universal screening of resistant organisms with tailored prophylaxis, though some have suggested that adopting this approach in high-risk patients, with appropriate clinical context and in conjunction with other prognostic indicators, may be beneficial [82].

### 4.3. Tailored Prophylaxis According to Institutional Data

While pre-operative screening for pathogenic organisms with subsequent individualized prophylaxis has demonstrated to have some efficacy, its application is limited by public health considerations such as prevalence, cost, and NNT and NNS. In contrast, the use of institutional data to guide prophylactic regimens has fewer downsides and has shown significant promise. 

This approach has produced especially favorable results in patients undergoing pancreaticoduodenectomy (PD), an operation with particularly high wound infection rates and that has been grouped under “abdominal procedures”, without consideration of biliary contamination, in most prophylaxis guidelines [82,83,84]. Donald et al. reduced their post-PD SSI rate from 32% to 7% by switching their prophylaxis from a guideline-adherent regimen to ampicillin–sulbactam after an internal review showed a high incidence of SSIs caused by resistant bacteria [85]. Similarly, Sano et al. and Tanaka et al. reduced their rates of post-PD SSI by greater than 25% by switching their prophylaxis regimens to align with the results of institutional culture surveillance data [86,87]. Kondo et al. conducted an innovative study in which they divided 116 consecutive PD patients into an early group and late group according to the date of surgery, with the early group receiving standard prophylaxis and the late group receiving prophylaxis according to susceptibility data isolated from SSIs in the early group [88]. The incidence of SSIs was 46.6% in the early group compared to 24% in the late group, and belonging to the late group was an independent negative risk factor for SSI. Other studies have shown similarly promising results, and a 2022 systematic review and meta-analysis of seven studies and 849 patients who underwent PD showed that targeted prophylaxis reduced the SSI rate from 42% to 21%, with the authors arguing in favor of this approach [89,90,91].

While each of these studies promotes tailored prophylaxis regimens prior to PD, it must be noted that doing so without incorporation of historical institutional data seems to be significantly less effective. For example, Stack et al. demonstrated organismal nonsusceptibility to standard prophylaxis in most of their patients’ hepatobiliary SSIs, but found that broadening coverage to include agents with activity against enterococcus or pseudomonas coverage did not significantly reduce the SSI rate [92]. Similarly, Fong et al. described SSI resistance rates of 53%, 69%, and 73% against the prophylactic regimens used in patients undergoing PD across three separate institutions [93]. Importantly, each hospital had remarkably different microbial characteristics, and certain prophylaxis regimens in one institution would have faced complete microbial resistance in another. In elective colorectal surgery patients, broadening coverage with stronger agents such as carbapenems or fluoroquinolones has not shown definitive benefits in reducing the SSI rate compared to standard regimens such as a cephalosporin with metronidazole [94,95]. 

### 4.4. Tailored Prophylaxis According to Individual Cultures

An extension of this approach is to tailor antibiotic prophylaxis based on the individual’s unique infectious history. Feldt et al.’s study would suggest a predictive benefit to an individual’s own prior SSI culture, but as far as we can tell, this has never been described. However, much can be learned from the studies of the prognostic value of an individual’s bile culture prior to HPB surgery. 

Bile is typically sterile in healthy patients but can become colonized or infected when bacteria migrate from the intestinal tract into the biliary system [96]. While this can be caused by malignant obstruction and subsequent biliary stasis, in patients undergoing PD, it is often caused by pre-operative manipulation of the biliary tract through procedures such as biliary drainage, decompression, or stenting. Intended to alleviate the physiological disturbances associated with biliary obstruction prior to undergoing surgery, these procedures have been shown to increase the risk of bile contamination and post-operative wound infections by as much as 80% [97,98]. Furthermore, they have been shown to induce polymicrobial infections and shift the biliary microbiome to be colonized with aggressive, resistant bacteria [99,100].

As a result, studies have investigated whether bile cultures, either taken pre-operatively through external conduit or more commonly intra-operatively after common bile duct ligation, have predictive utility in patients who have undergone biliary manipulation prior to PD. The results have varied. Howard et al. notes that while pre-operative biliary stenting in their patients did significantly increase the risk of infectious complications, just 42% of wound infections had bacteria that were also cultured from bile intra-operatively [101]. Cortes et al. demonstrated significantly increased infectious complications in patients with infected bile compared to sterile bile, but noted organismal concordance in just 49% of infectious complications [102]. Sandini et al. showed that just 35% of patients with resistant bacteria in their bile culture ultimately developed a post-operative infection [103], Maxwell et al. showed concordance rates between bile cultures and post-operative infection cultures of just 31%, and [104] Groen et al.’s cohort study and meta-analysis of 15 studies suggested that positive bile culture was not significantly associated with organ space infection or SSI [105]. Additionally, some studies note that even if intra-operative cultures do correlate with post-operative wound infections, there is not sufficient evidence to suggest that this increased infectious risk worsens post-operative morbidity and mortality [100,106]. 

In contrast, Povoski et al. found that 89% of patients with an intra-abdominal abscess and 95% of patients with wound infections had positive intra-operative bile cultures, and that the microorganisms isolated in bile were predictive of 100% of intra-abdominal abscesses and 69% of wound infections [107,108]. Sugawara et al. found that in patients with a superficial or deep incisional SSI, 98.6% had positive peri-operative bile cultures and 89% had the same microorganism isolated in the blood as in bile [109]. Itoyama et al. showed a 95% concordance between the bacteria collected in an intraperitoneal drain on post-operative day 3 and those collected in bile intra-operatively, and Limongelli et al. showed an 81% concordance between the bile culture and post-operative wound cultures [110,111]. Furthermore, several studies have associated positive cultures with a significantly greater risk of post-operative infectious complications and morbidity and have argued in favor of the predictive utility of bile cultures [93,103,111]. 

Some studies have also found a benefit to tailoring prophylaxis according to pre-operative bile cultures. In Sudo et al.’s study, for example, 34 patients underwent external biliary drainage pre-operatively, allowing for bile aspiration and culture with a subsequent tailored prophylactic regimen [100]. As expected, these high-risk patients had higher rates of biliary contamination, polymicrobial bile infections, and resistant organisms isolated in culture, but their infectious morbidity rate was not significantly higher than that seen in the low-risk group (13% versus 11%). Okamura et al. conducted a similar study of patients undergoing various HPB surgeries who were randomly assigned to receive either standard cefmetazole or targeted prophylaxis based on bile culture results collected from external biliary drainage [112]. The targeted prophylaxis group had a significantly lower rate of SSI compared to the standard prophylaxis group (43.5% versus 71.0%) without a meaningful increase in MDR pathogens in bile culture. Finally, in patients undergoing hepatectomy with biliary reconstruction, Makino et al. showed that pre-, peri-, and post-operative bile cultures have strong concordance rates with various post-operative infections, such as incisional SSI, organ/space SSI, bacteremia, and pneumonia [113]. The authors concluded that surveillance bile cultures taken over time can help predict and prevent not just SSIs but also seemingly unrelated infections such as pneumonia. 

### 4.5. Review of Tailored Prophylaxis

The aforementioned studies present a mixed picture. Pre-operative screening and prophylaxis tailoring for MRSA colonization or infection has been adopted due to the strong reductions in post-operative infections associated with such an approach. While similarly beneficial results have been seen with surveillance of other resistant pathogenic organisms, universal pre-operative screening recommendations for these organisms have not been issued, likely due to public health considerations of metrics such as NNT and NNS.

Similarly, tailored prophylaxis according to local and institutional data seems quite promising. Institutions have unique resistance patterns and SSI rates, and adapting prophylaxis based on reviews of these data seems sensible and more effective than simply broadening coverage. Yet, this approach is not a panacea, as it still fails to consider individual infectious history. Furthermore, given the known history of antimicrobial resistance, it seems likely that internal audits might need to be conducted every few years to “reset” prophylaxis regimens based on emerging resistance patterns.

Using a patient’s own prior culture data to individualize prophylaxis of course seems like the most specific approach, but it is limited by conflicting studies of prognostic efficacy, clinical utility, and the simple availability of sources to be cultured. Studies of organismal concordance between bile cultures and wound cultures have shown mixed results, and some clinicians have questioned whether even perfectly predictive bile cultures would be clinically useful, as most intra-operative cultures take 48 h to result, by which time routine 24 h post-operative antibiotics have already been given and some infections have already begun to brew. On the other hand, many infections arise >5 days post-operatively, in which case predictive bile cultures could help direct accurate, timely therapies, and in the minority of patients who do undergo external biliary drainage, accurate bile cultures taken pre-operatively could help direct individualized prophylaxis. It also must be noted that most non-HPB surgeries simply lack fluids that could be cultured for predictive or prognostic benefit. Additionally, studies of organismal concordance require accurate wound cultures, and many surgeons today treat their post-operative infections empirically without culturing or assessing the organism responsible.

### 4.6. The Pathogenesis Underlying Recurrent Post-Operative Infections

One of the difficulties in adopting tailored pre-operative prophylaxis is the simple fact that we lack an understanding of the underlying pathogenesis between a prior SSI and a subsequent SSI. Most ideas regarding this pathogenesis are speculative.

The “weak host” theory suggests that certain patients are susceptible to SSIs through undetectable innate characteristics, such as subclinical immunosuppression, which only becomes apparent after surgical stress [44]. Several patient-related risk factors for post-operative infections are well-established, including older age, obesity, diabetes, smoking, and chronic diseases. While Feldt et al. noted no demographic differences in those who developed an SSI after the second operation compared to those who did not, it is entirely possible that there are underlying risk factors that were not accounted for, or that we have yet to uncover. 

Antibiotic resistance could certainly be implicated. It has long been known that the most common source of an SSI is the patient’s own flora, and that antibiotic use can select for resistant commensal organisms that ultimately render future antibiotics less effective [114,115]. Thus, it is plausible that the treatment of an initial SSI leaves patients colonized with resistant bacteria that make subsequent prophylaxis regimens less effective. Several studies described in this review certainly reflect that. If true, patients undergoing multiple operations may experience a cascade effect whereby each round of antibiotics for an SSI selects for increasingly resistant bacteria that are less and less responsive to routine prophylaxis in subsequent operations. 

Perhaps prolonged, lingering bacterial colonization following a post-operative infection is responsible. Several of the earliest studies of recurrent infections in hernia repair patients described this. However, these studies described recurrent infections within the same tissue site and within a somewhat protracted amount of time, compared to Feldt et al.’s findings that linked post-operative infections together that occurred in unrelated surgeries and often many months or even years apart. Nonetheless, it is possible that some bacteria can survive the “curative” antibiotics prescribed for a post-operative infection, lingering in the body until the stress of a subsequent surgery allows them to proliferate and cause another infection. 

Another theory has looked to sepsis survivors, who have poorer long-term outcomes, including an increased risk of subsequent infection and overall mortality, compared to matched cohorts [116]. This appears to be the case even 10 years after the septic episode, in previously healthy individuals, and in comparison to patients who have suffered similarly serious bodily insults that were not septic [117,118]. While the pathogenesis of this phenomenon is poorly understood, newer evidence has implicated the gut microbiome. When healthy, the gut microbiome modulates the adaptive immune system and helps shield the body from traumatic stressors [119]. But a single infectious trigger, such as septic bacteremia, along with curative courses of antibiotics, can create a pro-inflammatory “pathobiome” that leaves the host with decreased microbial diversity, immune-suppressed, and less able to respond to subsequent traumatic events [120,121]. It is plausible that post-operative SSIs, especially those in the gastrointestinal tract that disrupt the gut’s epithelial integrity, can lead patients to similarly vulnerable states in which they are more likely to succumb to an SSI following surgery [114]. 

There may be something to learn from the link between post-operative infections and worse outcomes in various malignancies. Indeed, while the evidence is quite conflicting, some studies have shown that a post-operative infection after an oncological resection is an independent risk factor for earlier cancer recurrence and even decreased survival [122,123]. While this phenomenon is poorly understood, investigators have suggested that post-operative bacterial infections can induce systemic inflammatory syndromes that downregulate tumor-specific immune responses and anti-inflammatory cytokines, ultimately increasing the likelihood of dormant tumor cell proliferation and recurrence [122,123]. It is plausible that a similar downregulation of protective immune cells from post-operative infections can leave the patient susceptible to subsequent post-operative infections.

While each of these theories is certainly possible, it is clear that our understanding of the link between a prior infectious history and subsequent post-operative infections is still limited. To develop a better understanding, future studies should follow patients over extended periods of time as they undergo multiple surgeries. Those who develop post-operative infections should have their infections cultured and tested for susceptibility to both the curative antibiotics chosen as well as the pre-operative prophylaxis regimen used. The development of non-SSI infections should also be recorded and evaluated to determine any concordance with post-operative infections and evaluate for subclinical immunosuppression. If equipoise can be achieved, randomizing patients to standard prophylaxis versus prophylaxis tailored to prior infections could provide prospective evidence of the benefits suggested in this review. 

Our review has several limitations. The first is that the studies used for this review are heterogeneous in their patient population, hospital setting, geographic setting, disease of interest, and physician prescribing practices. This heterogeneity makes it difficult to make sweeping claims about the utility of tailored prophylaxis but bolsters the argument for future studies incorporating larger patient populations, standardized antibiotic regimens, long-term follow-up, and well-defined end points. The second limitation is the scarcity of reliable underlying data. Because many clinicians do not culture or report the organismal content of their patients’ SSIs, we lack a deep understanding of the common resistant profiles of the organisms that cause post-operative infections. This, in turn, makes it difficult to study tailored prophylaxis because there are simply insufficient culture data to draw from when making decisions about prophylaxis regimens. Finally, connecting a prior infectious history with an increased risk of a future post-operative infection is itself fraught with challenges in that it demands connecting two events at different points in time and in different circumstances. Over one’s life, an individual might move locations, change health insurance, see different providers, develop a variety of conditions, and undergo a myriad of other life changes, thereby making it difficult to isolate the exact role that a single post-operative infection may play in contributing to a patient’s future risk of infectious complications following surgery that may or may not be related to the prior operation. Much more research is needed to better understand this important topic.

## 5. Conclusions

Individualized pre-operative prophylaxis may offer a novel, appealing method of reducing a patient’s risk of post-operative infection. While some studies support this approach, the predictive and prognostic value of a patient’s prior infectious history remains controversial. Further research must be performed to better elucidate the findings described in this review. 

## Data Availability

Not applicable.
